# A Bio-inspired Motivational Decision Making System for Social Robots Based on the Perception of the User

**DOI:** 10.3390/s18082691

**Published:** 2018-08-16

**Authors:** Marcos Maroto-Gómez, Álvaro Castro-González, José Carlos Castillo, María Malfaz, Miguel A. Salichs

**Affiliations:** Department of Systems Engineering and Automation, Universidad Carlos III de Madrid, 28911 Madrid, Spain; acgonzal@ing.uc3m.es (Á.C.-G.); jocastil@ing.uc3m.es (J.C.C.); mmalfaz@ing.uc3m.es (M.M.); salichs@ing.uc3m.es (M.A.S.)

**Keywords:** decision making, social robots, HRI, machine learning, motivation, drives, homeostasis, RGB-D, user detection

## Abstract

Nowadays, many robotic applications require robots making their own decisions and adapting to different conditions and users. This work presents a biologically inspired decision making system, based on drives, motivations, wellbeing, and self-learning, that governs the behavior of the robot considering both internal and external circumstances. In this paper we state the biological foundations that drove the design of the system, as well as how it has been implemented in a real robot. Following a homeostatic approach, the ultimate goal of the robot is to keep its wellbeing as high as possible. In order to achieve this goal, our decision making system uses learning mechanisms to assess the best action to execute at any moment. Considering that the proposed system has been implemented in a real social robot, human-robot interaction is of paramount importance and the learned behaviors of the robot are oriented to foster the interactions with the user. The operation of the system is shown in a scenario where the robot Mini plays games with a user. In this context, we have included a robust user detection mechanism tailored for short distance interactions. After the learning phase, the robot has learned how to lead the user to interact with it in a natural way.

## 1. Introduction

Social robotics is an emergent field which is currently in vogue. Many researchers anticipate the spread of robots coexisting with humans in the near future [1,2]. This requires a considerable level of autonomy in robots. Moreover, in order to provide a proper and natural interaction between robots and humans without technical knowledge, these robots must behave according to the social and cultural norms. This results in social robots with cognitive capabilities that can be inspired by biological organisms such as humans or animals.

When talking about decision-making systems (from now on DMSs) in robotics, we refer to the capacity of a robot to make its own decisions autonomously, without the control of a human “operator”. In this work we explore how living beings make decisions and, taking inspiration from nature, we propose to extend the autonomy of a social robot by implementing a biologically inspired DMS.

Pfeifer et al. [3,4] stated that the goal of biological inspiration in robotics is to understand the behaviors of animals, transfer them to robots, and obtain some desirable properties from biological organisms. Following this idea, our DMS is based on biological concepts, such as drives, motivations and self-learning. According to psychological theories, drives are deficits of internal variables or needs (e.g., energy) and the urge to correct these deficits are the motivations (e.g., survival). Following a homeostatic approach, the goal of the robot is to satisfy its drives maintaining its necessities within an acceptable range, i.e., to keep the robot’s wellbeing as high as possible. In order to do so, the learning process provides the robot with the proper behaviors to cope with each motivation in order to achieve the goal.

In this paper we aim at presenting our DMS that uses bio-inspired concepts to foster the human-robot interaction and tailors the robot’s behavior to the people around the robot. The operation of the DMS has been evaluated with a user who interacts with the robot, which has two motivations: *Social* and *Relax*. We have observed how the robot’s behavior fosters the interaction while simultaneously keeps its wellbeing as high as possible. In this case, the robot learns a behavior (or policy of actions) to execute when this user is around. In the case of considering different users, it is likely that each person behaves differently when interacting with the robot and therefore the robot would need different policies for each user.

The scenario where this DMS is applied involves a real interaction between a user and the robot, so its perceptual capabilities are crucial. In this paper the robot needs to perceive the location of the user and this information is then used by the DMS to select the actions to execute.

In Section 2 we describe how animals make decisions and present the most relevant theories about the reasons to behave in a particular way. These theories have driven our approach. Following, Section 3 presents other works where bio-inspired DMS have been used in robotics. After that, Section 4 describes how those biological concepts (presented in Section 2) have been modeled in a decision-making system to be implemented on our social robot Mini. Section 5 presents the user detection system that has been developed for one-to-one human-robot interactions. In Section 6, we evaluate our DMS in a scenario where the robot Mini plays with a user, and the results are presented in Section 7. Finally, we conclude the paper summarizing the contributions and pointing out the challenges and the next steps (Section 8).

## 2. The Origin of Behavior

All animals are endowed with systems or mechanisms which are in charge of selecting the behaviors or actions to execute at each instant according to specific reasons. In animals, behavior is considered as a manner of acting due to certain circumstances in order to achieve certain goals. The brain is responsible for all kinds of behavior arrangements, from seeking food to falling in love. Certain brain neurons (electrically excitable cells) communicate with hundreds of thousands of cells around the whole body to orchestrate their functions and, as a consequence, behaviors arise. Then, behaviors can involve many organs (heart, liver, lungs, kidneys, etc.), and without them, all behaviors would fail.

When animals make decisions, these can be *innate* or *learned*. Innate decisions are inherited and are species dependent. Some authors [5] consider them as instincts which are fundamental to the development of the individual. For instance, a baby animal already knows that it has to suckle from its mother. On the other hand, learned decisions consider the past experiences. As a result, when a decision has been learned, the behavior is guided by past experiences [6]. Then, decisions result from a combination of both innate and learned, and they coexist side-by-side.

Veldhuis affirms that, such as in other high cognitive processes, decision making has a dual-processing perspective: *conscious* and *unconscious* [6]. Therefore, there are two systems of decision making which, somehow, are related: (i) System 1: unconscious, fast, automatic (or reactive), and high capacity decision (e.g., intuitive decisions). Prior knowledge is used to form a response. In this system, decisions are *involuntary*; and (ii) System 2: the highly conscious, slow, and deliberative decision (e.g., reflective decisions). It could happen that this system does nothing (it does not have any effect), so the unconscious responses keep on working, or it inhibits the unconscious responses for developing a more conscious strategic thinking. These kinds of responses are *voluntary*.

According to Veldhuis, in general, decisions are made unconsciously but, when a novel event happens, the deliberative conscious system takes over. This assumption implies that any deficit in System 1 greatly affects our decision-making capacity. Without the unconscious decision-making system, all information has to be processed by the deliberative system. Due to its low capacity and slow processes, it results in very slow decision making and a potential loss of information.

### 2.1. Homeostasis

Animals have to carefully control some internal conditions. For example, mammals live under tight conditions of body temperature and blood pressure, volume and composition. These variables must keep their values in a narrow range. The hypothalamus adjusts these levels in response to changes coming from the external environment. This regulatory process is called *homeostasis*: the maintenance of the body’s internal environment within a narrow physiological range [7]. Homeostasis was discovered by Claude Bernard in the middle XIX century when he observed that the body variations had as an objective to give the stability back to the body. According to the homeostatic approach, the human behavior is oriented to the maintenance of the internal equilibrium [8].

An example of this tendency towards internal stability can be easily observed in temperature regulation. Cells properly work at 37 °C and variations of more than a few degrees are catastrophic. Precise cells belonging to the whole body perceive modifications on body temperature and respond to this situation. In an extremely cold situation (e.g., being naked in the North Pole), the brain sends commands to generate heat in the muscles (shivering), to increase tissue metabolism, and to keep blood as far as possible from external cold surfaces of our skin in order to maintain internal warm (you turn blue). In contrast, if we are in a sauna, the brain activates cooling mechanisms: blood is moved to the external tissues where heat is radiated away (we turn red) and the skin is cooled by evaporation (sweating).

In order to maintain the homeostatic balance, the whole body responds with voluntary and involuntary behaviors. All behaviors are orchestrated by the brain which reaches organs by means of the nervous system. The combination of the nervous system and the somatic motor system originates different behaviors.

### 2.2. Motivated Behavior

The key question at this point is why behavior occurs. Clark Hull postulated in 1943 his drive-reduction theory [9]. This is one of the oldest theories about drives. Hull suggested that privation induces an aversion state in the organism, which is termed drive. According to his theory, the drives increase the general excitation level of an animal and they are considered as properties of deficit states which motivate behavior.

He stated that all the behaviors happen as the result of physiological needs, the drives. According to his theory, the reduction of drives is the primary force behind motivation [10]. He based his theory on the concept of homeostasis, i.e., the body tends to maintain certain internal balance and actively works for it. Behavior is one of the resources the body has for achieving it. Considering this approach, Hull postulated that all motivations come up due to biological needs, which Hull referred to as drives (thirsty, hunger, warmth, etc.). Thus, a drive produces an unpleasant state that has to be reduced by means of the corresponding behavior (e.g., drink when we are thirsty or close the windows when we are cold).

This reduction of drives serves as a reinforcement for that behavior. In the future, when the same need arises, the reinforced behavior will be more likely repeated. In other words, when a stimulus and a response provoke a reduction in the need, the probability that the same stimulus causes the same response increases [11].

However, many years later the Hull’s theory started to fall out of fashion due to many criticisms [12]. First, Hull’s theory does not consider secondary reinforces. Primary reinforcers satisfy survival needs such as food, shelter, or safety. Secondary reinforcers are those that can be used to obtain primary reinforces. Some examples could be money, praises, or grades in school. Moreover, this drive-reduction theory does not explain the behaviors that are not related to biological needs and therefore do not reduce drives. Why do people eat when they are not hungry? Why do people skydive? This theory does not answer these questions.

Later, other researchers started to tackle the not explained questions in the Hull’s drive-reduction theory. Kandel et al. described a motivation as an inferred internal state postulated to explain variability in behavioral responses [5]. Motivational states represent urges or impulses that impel animals into action. Initially, motivations were linked to bodily needs such as energy or temperature regulation (classical homeostatic drives). However, other non-physiological needs are well-accepted as motivations too, e.g., curiosity or sex. However, all these needs are referred to as drives because they involve arousal and satiation. The concept of *drive* is postulated in order to explain why observable stimuli in the external environment are not sufficient to predict behaviors. For example, sometimes food can stimulate feeding, but at other times, it results in indifference or even rejection. E.g., when you walk down a street and see chocolate, it can provoke the “need” to eat chocolate. In contrast, after a big meal, the perception of more food activates a denial reaction.

The motivations can be seen as a driving force of behaviors. However, just motivation does not guarantee a behavior but it modulates the behavior and affects its probability of happening. Besides, several motivations may interfere with each other, for example, the need for food versus the need for sleep. The word motivation derives from the Latin word *motus* and indicates the dynamic root of the behavior, which means the internal, more than external, factors that urge to action [13]. Sometimes, motivational states can be explained as a compendium of internal and external stimuli. Hence, *motivation* can be presented as a complex reflex under the control of multiple stimuli, some of them internal [5]. Hull [9] also proposed the idea that motivation is determined by two factors. The first factor is the drive. The second one is the incentive, that is, the presence of an external stimulus that predicts the future reduction of the need. For example, the presence of food constitutes an incentive for a hungry animal.

The following example will clarify the ideas previously introduced. When a person is cold, dehydrated, and depleted of energy, the proper responses automatically come through. This person shivers, his blood is moved away from the body surface, urine production is inhibited, body fat reserves are mobilized, and so on. However, the most effective and fastest way to correct the disruptions is to look for a warm place, to drink water and to eat. These are *motivated behaviors* generated by the Somatic Motor System (formed by the skeletal muscles and the nervous system that controls them) and incited to occur by the hypothalamus [7].

Hypothalamus and related structures receive information from the internal environment and they directly act over the internal environment (if you are cold, your body temperature is directly kept constant by peripheral vasoconstriction). Other hypothalamic neurons are in charge of operating indirectly over the internal environment, by means of the SMS acting in the external environment (if you are cold, you can turn the heat on). Both indirect and direct homeostasis can work in parallel. Besides, the already mentioned System 1 and System 2 can be observed in the previous example; vasoconstriction is a unconscious and involuntary reaction which can be placed at System 1; turning the heat on corresponds to System 2 where conscious, voluntary actions are made.

The intensity of a motivation depends on several factors. Considering hunger is the motivation to eat, it depends on how much you ate the last time, what kind of food, and how long it has been since. After we finish eating and the digestive process has begun, the need for energy is inhibited due to satiety signals. These satiety signals slowly dissipate until the need to eat again takes over. That means that just after eating, satiety signal soars; then, it slowly vanishes until the next ingestion of food, when it rises again. In general terms, drives, in the sense of needs or deficiencies, lead the regulatory process of motivations. Drives vary according to several signals and parameters. However, the presence of incentives (external stimuli), can alter the course of motivations and/or drives.

#### Cognitive Aspects of Motivations

After understanding the physiological aspects of the motivation of behaviors (especially those that are basic to survival), it seems that humans are ruled by hormones whose secretion is activated by neurons all over the body. However, researchers clarify that one of the main advantages of human evolution is our capacity to exert cognitive control over our more primitive instincts.

Motivational behaviors are not only attached to physiological needs. For example, curiosity does not appear to be commanded by any physiological shortage. Particularly in humans, learned behaviors and pleasant feelings can prevail against bodily signals. This is the case when a person feels the need to go to the toilet but he is attending an important meeting and he cannot leave the room.

In psychological terms, there are two points of view about motivated behaviors:

**Hedonic** People exhibit a behavior because they *like* it, it feels good so people do it ( e.g., the smell, taste, and sight of food, and the act of eating itself are pleasant). Pleasure serves as a hedonic reward.
**Drive reduction** People *need* to behave in a certain way in order to satisfy a drive (e.g., animals eat because they are hungry and *want* food).

Both approaches seem to be complementary (we drink what we like) but, apparently, “liking” and “wanting” are controlled by different circuits in the brain [7]. Conversely, other researchers [5] identify three factors as motivated behavior regulators:

**Ecological constraints** Behavior patterns have been shaped by evolutionary selection. Ecological context is analyzed by cost-benefit functions. Feeding behavior includes the cost of searching and procuring food and the benefits of the energy obtained from the nutrient intake.
**Anticipatory mechanisms** Clock mechanisms activate physiological behavioral responses before the need or the deficit in the tissues occurs. Therefore, homeostasis often anticipates deficits.
**Hedonic factors** Pleasure is an undoubted factor in the control of the motivated behavior of animals. Frequently, humans give up some need in order to obtain pleasure by satisfying others. For example, people go on a diet because they want to look more attractive. It gives the idea that pleasure mechanisms are concerned with reward and reinforcement on learned behavior.

The ecological constraints, the anticipatory mechanisms [5], and the drive reduction [7], somehow, are all related to physiological needs. The hedonic factor in motivated behavior is clearly identified in both approaches. Since pleasure is an evident element in motivated behavior, researchers have studied how it is evoked. Olds [14] discovered pleasure areas on some animals’ brains. Later, Deutsch and Howard [15] found that stimuli of pleasure areas on the brain originate reinforcement independently of the drive state of the animal. In contrast, regular stimuli just function as a reward in particular states (food is considered as a reward just in hungry animals). Successive studies have shown that pleasure areas in the brain are involved in initiating some complex behaviors such as feeding and drinking. Apparently, the hypothalamus is one of the areas that produces reward and several transmitters seem to take part.

In this work, our DMS considers motivated behaviors oriented to the drive reduction in order to maintain homeostasis. These behaviors are learned by experiences and executed automatically (i.e., they are selected without deliberation). Other features, such as innate conscious behaviors or hedonic factors in motivated behaviors, will be included in future works.

## 3. Bio-Inspired DMS in Robotics

Some of the previous ideas existing in animals have already been applied to robotics. In the late 1990s, researchers started to incorporate bio-inspired DMS in robots. One of the first works was Cathexis [16], an architecture developed by Velasquez where several behaviors compete for the control of the robot. The winning behavior applied its *expressive* component (poses, expressions, or utterances) and its *experiential* component that altered the drives and motivations of a robot.

A few years later, Arkin et al. introduced a model where the motivations were related with emotions (fear) and physiological needs (hunger and sex) [17]. The highest motivation activates the corresponding behavior just when the associated external stimulus is present. Arkin continued this line with a new work where both external and internal needs were included as part of a robotic behavior system [18]. In that work, the robot AIBO selected its behaviors with the aim of regulating its needs. Later on, Arkin and Stoytchev incorporated circadian rhythms in the evolution of the robot’s need in a way that their values depended on the time of the day [19].

Cañamero proposed to link the motivations with the survival of the agent [20] and advocated for the competition of motivations and, considering the highest one, the robot behavior is selected in order to satiate the most urgent need [21]. In contrast with Cañamero’s competition of motivations, Breazeal proposed a competition of behaviors that resulted in one behavior taking over [22]. The relevance of each behavior is computed based on internal variables and external factors from the environment. At the same time, Parisi presented simulated robots with physiological needs (e.g., food and water) and he stated the importance of these internal factors to achieve robots that behave naturally [23].

Lately, researchers have begun to apply homeostatic systems in order to promote the social interaction between robots and humans. Vouloutsi et al. presented an android that was endowed with various drives intended for obtaining a socially accepted behavior [24]. In order to achieve it, the android had multiple goals that were coupled together with the drives. Drives and goals motivated the robot’s behavior and, in addition, were used to evaluate the action outcomes. In this line, Cao et al. presented a behavior system where drives competed to select the next action in a human-robot interaction (HRI) scenario [25]. In this system each drive is coupled with a repertoire of actions and, among them, the next action is selected in case the associated drive is the winner. The system was applied to a scenario where children, a therapist, and the robot Nao interacted. Recently, Hieida et al. have combined a homeostatic system with unsupervised learning with the aim of allowing the robot to learn behaviors that consider some social aspects [26].

In our work, we are in line with Cañamero’s approach where motivations compete to lead the robot’s behavior. However, we consider a robot that has to learn from scratch how to interact with a user. Thus, similarly to Hieida et al., we endow our robot with a learning mechanism that allows it to select the most appropriate actions according to the user.

## 4. A Motivational Decision Making System for a Social Robot

In this section, we present our DMS, which is inspired by many of the concepts already observed in nature and presented in Section 2. In our case, the DMS is aimed at allowing a social robot to decide what to do at every moment without predefined goals. The objective of the robot is to *feel good*, in the sense that it has to keep its needs within an acceptable range. Nevertheless, the way to achieve this goal is not defined.

In this DMS, the autonomous robot has certain needs (drives) and motivations. The goal is to survive by keeping all its drives satisfied. For this purpose, the robot must learn to select the right action in every state in order to maximize its wellbeing. The robot’s internal state is configured by several variables, which must be at an ideal level. When the value of these variables differs from the ideal one, an error signal occurs: the drive. These drives constitute urges to act based on bodily needs related to self-sufficiency and survival [21]. In this approach, the drives are considered as the needs of the robot. The ideal value for a drive is zero, which corresponds to the lack of need. As time goes by, the drive increases until it is reduced or satiated (reset to zero).

Following the ideas of Hull [9] and Balkenius [27], the intensities of the motivations of the robot are modeled as a function of its drives and some external stimuli. The motivational states represent tendencies to behave in particular ways as a consequence of internal (drives) and external factors (incentive stimuli) [28]. In order to model the motivations of the robot, we are inspired by the ideas of the Lorentz’s hydraulic model of motivations [29]. In Lorenz’s model, the internal drive strength interacts with the external stimulus strength. External stimuli are perceptions coming from the environment that alter the tendency to act, that is, the motivations to behave in one way or another. For example, in animals, the smell of a tasty food increases the motivation to eat. Therefore, if the drive is low, then a strong stimulus is needed to trigger a motivated behavior. If the drive is high, then a mild stimulus is sufficient [8]. If the drive or the stimuli separately are strong enough, a behavior can be induced without the influence of the other. The general idea is that we are motivated to eat when we are hungry and also when we have food in front of us, although we do not really need it. In nature, a weak stimulus (e.g., spoiled food) but a strong motivation (e.g., starving) may result in the same behavior as a strong stimulus (e.g., chocolate cake) but weak motivation (e.g., full stomach) [30]. Therefore, the intensities of the motivations are calculated as shown in Equation (1)

(1) $$\begin{array}{c} \mathrm{If}\;D_{i}<L_{d}\;\mathrm{then}\;M_{i}=0\\ \mathrm{If}\;D_{i}\geq L_{d}\;\mathrm{then}\;M_{i}=D_{i}+w_{i} \end{array}$$

where $M_{i}$ is a particular motivation, $D_{i}$ is the related drive, $w_{i}$ corresponds to the related external stimuli, and $L_{d}$ is called the activation level. Motivations whose drives are below their respective activation levels will not be able to lead the robot’s behavior.

According to Balkenius [27], all excited motivational states cannot be allowed to direct the robot at once since this would generate incoherent behaviors. In his opinion, this problem cannot be handled solely by behavioral competition but must be resolved at an earlier stage of processing. The solution proposed is a motivational competition, as Cañamero also put forward in [31]. Therefore, in this approach, once the intensity of each motivation is calculated, they compete among themselves for being the dominant one. The motivation with the highest value, and which drive is over its activation level (Equation (1)), is considered the **dominant motivation**, and it determines the internal state of the robot. If the drive is below the activation level, it does not compete for being the dominant motivation.

When none of the drives is greater than its activation level $L_{d}$, it happens that there is not a dominant motivation. This occurs when all drives are satisfied or, at least, their values are close to their initial values of zero. This implies that the robot’s wellbeing is very high, close to the ideal wellbeing. The lack of dominant motivation means that all needs are not high enough to induce the robot to act, so it is in a pleasant state. This is interpreted in such a way that a particular behavior that reduces the drive related to the dominant motivations is not necessary.

The state of the robot is a combination of the inner and external state. The inner state, as has just been explained, is determined by the dominant motivation of the robot. The external state is defined by its relation to every object in its environment. The action selected at each moment will depend on the state of the robot and the potential actions since the external state restricts the possible actions. In humans, for example, we cannot eat if we do not have food. It is important to note that initially, the robot does not necessarily know the consequences of its actions nor the reinforcement that it will receive. For instance, the robot does not know that after recharging its batteries, its level of energy will be high. The robot just has the knowledge about which actions can be executed in every state.

**Figure 1:**
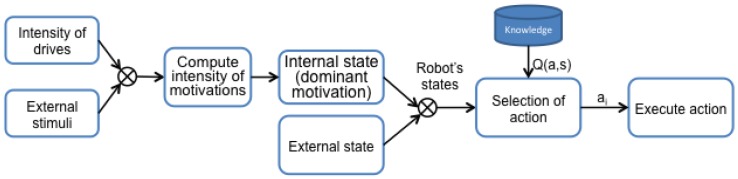
Steps followed by our DMS.

The robot makes decisions based on the value of executing each action in the current state, represented as $Q(a_{i},s_{t})$. In other words, $Q(a_{i},s_{t})$ represents how good it is to execute the action $a_{i}$ in the moment *t* when the robot is in the state $s_{t}$. The whole process can be seen in Figure 1.

When the robot does not count on any previous knowledge, all values $Q(a_{i},s_{t}),\forall i$ are equal. That is, all actions are equally good. This means that the robot does not know in advance which actions are the best ones for dealing with the dominant motivation. The robot has a repertory of actions and they can be executed depending on the relation of the robot with its environment, i.e., the external state. For example, the robot will be able to interact with people as long as it is accompanied by someone.

If we want to provide our robot with *innate behaviors*, we can provide higher values for certain actions in certain states. This can be seen as inherited knowledge or instincts and our robot will likely decide to execute these actions when it is in those particular states. For instance, similarly to baby animals that know that they have to suckle from their mothers when they are hungry, we can provide a high value to the action *recharge batteries* when the level of energy is low and, thus, ease the survival of our robot.

Besides, *learned behaviors* are achieved by learning mechanisms which update the values $Q(a_{i},s_{t}),\;\forall i,s$. Through the learning process, the robot learns what action is the best in every situation. In our case, the robot has to learn by itself the policy of behavior considering a user. We have decided to use Reinforcement Learning in our system, in particular the well-known Q-learning technique [32]. In this case, the reward signal has been determined as a function of the robot’s wellbeing, which indicates the degree of satisfaction of its internal needs. The robot’s wellbeing is defined in Equation (2), where $D_{i}$ corresponds to the actual value of each drive and $Wb_{ideal}$ is the highest possible value of the wellbeing, i.e., when all drives are satiated.

(2) $$Wb=Wb_{ideal}-\sum_{i}D_{i}$$

The reward signal has been defined as the variation of the robot’s wellbeing (ΔWb) and, therefore, the robot will learn to maximize that variation; in other words, the robot learns which action is the best in terms of its wellbeing. ΔWb is calculated as the current wellbeing minus the wellbeing in the previous step ($\Delta Wb=Wb_{t}-Wb_{t-1}$).

## 5. User Detection

Our DMS relies on accurate user detection in a 1-to-1 HRI scenario. Therefore, the robot must detect the location of the user to determine its state in relation to it. More specifically, the robot was situated on a table, as will be explained in Section 6, so the user had to be close and seated in front of it to interact. For this reason, we followed an approach based on RGB-D information that is able to detect when a user is in front or near the robot, away from the robot, or when there is no user in the scene. For this purpose, we used the RGB-D sensor embarked in the robot, a Softkinetic DS325 camera, with a depth range from 0.2 to 1.5 m. This device possesses a wide field of view of 74 and 58 degrees for the horizontal and vertical dimensions respectively, at 320 × 240 3D points resolution. The colour sensor has a resolution of 720p at 30 frames per second (Softkinetic DS325 datasheet https://www.sony-depthsensing.com/Portals/0/Download/WEB_20120907_SK_DS325_Datasheet_V2.1.pdf).

**Figure 2:**
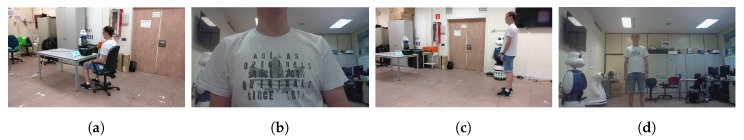
User standing in front of the robot. At a short range, the face detection cannot be applied since the head of the user is not visible in the image. At a medium and long range, the face can be detected but the depth stream cannot be used due to its limited range, 1.5 m.

The algorithm uses (i) colour information to locate the users’ face in the video stream (face detection module) at a medium and long range (limited by the camera resolution), and (ii) depth information in order to detect the body to complement the face information or in cases in which the face is not present in the image (body detection module) at a short range. It is important to remark that when interacting at a short range with a robot, the whole body is not usually visible and, depending on whether the user is standing or sitting, the head may fall out of the camera field of view. Figure 2 shows how the user is seen by the robot at a short and long range.

### 5.1. Body Detection

Since our robot integrates a static RBG-D camera, some well-known preprocessing techniques to enhance the detection process can be applied. The first step is intended to discard those 3D points that correspond to static objects in the field of view of the camera.

Therefore, a *Movement Filter* is applied to an input point cloud, $P^{t}$, where each $p_{i}^{t}=\left\{x_{i},y_{i},z_{i}^{t},grey_{i}^{t}\right\}$ corresponds to a point defined by its *x*, *y* and *z* coordinates plus an extra field, grey, that corresponds to the depth codified as grey values. Additionally, another reference point cloud, $P^{t-1}$, stores those points associated to movements in a previous time step defined as $p_{i}^{t-1}=\left\{x_{i},y_{i},z_{i}^{t-1},grey_{i}^{t-1}\right\}$. By comparing these differences in the points grey level between these two point clouds, a movement mask, *M*, can be calculated where movements corresponds to changes in the grey level of the points, $m_{i}$, setting the mask point to the maximum value if the variation is greater than a determined threshold, τ, or decrementing such mask point otherwise. Finally, those points that are 0 (no movement detected for a period of time) in the mask are used to null the points in the initial input point cloud, $P^{t}$, to obtain those regions in the point cloud in which movement has been detected, $P_{M}^{t}$.

**Figure 3:**
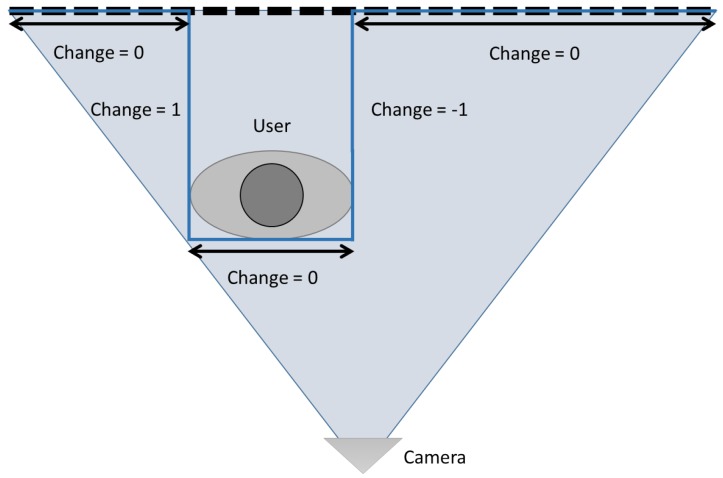
Depth changes produced by a user in the image.

Traditional techniques for human body detection usually rely on a complete (or almost complete) view of the body. In the case of close interaction with humans, this is not possible as the body exceeds the camera field of view (see Figure 2b). For this reason, our approach follows a different strategy that consists of finding shapes that could correspond to the features of human torsos. The first step is to locate in the point cloud those sudden changes in the z axis that could correspond to users in the scene. For this purpose, a change matrix, $C^{t}$, is defined with the same height and width as the input point cloud (in pixels) and the value of each position, $c_{i}^{t}$, is calculated depending on a change threshold, $\theta_{change}$. This threshold was set to 0.3 m, a conservative value taking into account the average male and female chest depth, 28 and 25 cm, respectively [33]. If the depth variation between two consecutive pixels, $z_{i}-z_{i-1}$, is lower than $-\theta_{change}$, $c_{i}^{t}$ is set to 1, 0 if the difference is located between $\left[-\theta_{change},\theta_{change}\right]$, and −1 if the difference is greater than $\theta_{change}$. Next, those regions between change zones are measured to check if they are wide enough as to be considered candidates to human torsos (see Figure 3). Since $C^{t}$ is a matrix containing pixel-level information, a minimum width of 30 pixels is established to filter out small volumes that could result in false positives.

### 5.2. Face Detection

For long-range detection we followed a simple approach. In our interaction scenario, the faces of the users interacting in front of the robot fall out of the camera field of view. Therefore, the body detector provided reliable information for short-range detection. Conversely, users further away from the robot could be detected in the RGB stream but not in the depth one. Thus, we integrated a face detector based on the well known algorithm of Viola-Jones [34].

The first step of this algorithm is extracting HAAR features, a set of descriptors that involve grouping image pixels within rectangular areas and exploit some properties common to human faces (e.g., the eye region is darker than the upper-cheeks, or the eyes are darker than the nose bridge). These features are then input into an AdaBoost algorithm that tries to build an accurate classifier as a linear combination of weighted simpler classifiers, each one trained for a specific HAAR feature. Finally, face detection is performed by executing those simple classifiers one after the another and, if at some point, a classifier detects that its feature is not detected, the region is rejected.

### 5.3. Merging Body and Face Detection

After performing both body and face detection, this component unifies the results and sends them to the decision-making system. The last step of our user detection algorithm consists of applying a temporal aggregation and a set of rules to decide what mechanism has detected a user. This process considers a window of size 15, counting the number of body detections, *b*, and face detections, *f*. The possible outputs from this component are three:

- *User is near*, if the number of body detections within the window is greater than two thirds of the window size and the number of detected faces is lower than one third.
- *User is far*, if the number of face detection within the window is greater than two thirds of the window size and the number of detected bodies is lower than one third.
- *User is not detected*, if the number of body and face detections is lower than one third of the window, respectively.

This information will serve as an input for the decision-making process to asses the state of the robot in relation to the user, which is called the external state (see Section 6.1.2).

## 6. Evaluation

The already presented DMS has been evaluated in a scenario where one robot interacted with one person in order to play educational games. The robot had to learn the proper policy of behavior in order to maximize its wellbeing.

**Figure 4:**
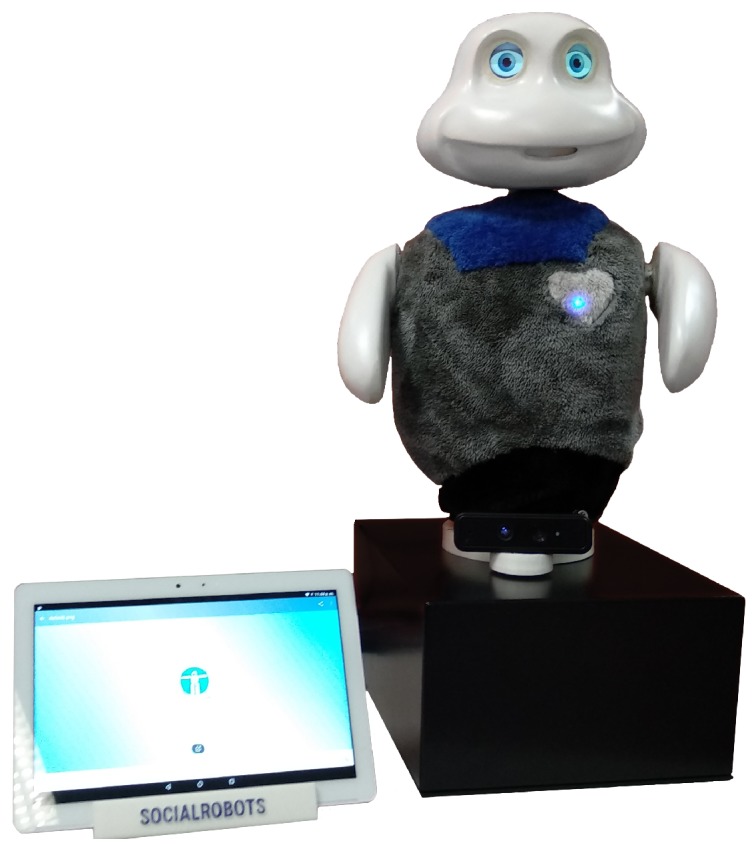
The social robot Mini.

In our work, we have used the robot Mini (Figure 4). Mini is a desktop robot which is able to perform a repertoire of cognitive exercises and educational games to stimulate and improve the interaction with users. In order to facilitate the interaction with the user, Mini has several touch sensors placed on different parts of its body, a microphone to obtain the user’s utterances, and an RGB-3D camera placed on its base to perceive objects and users around the robot. A Voice Activity Detection module is used to identify when the received audio signal corresponds to voice [35]. In relation to the actuators, Mini has five servomotors (one on its base, one per arm, and two on the head), several RGB LEDs on its body (one simulating a beating heart, two for the cheeks, and an 8-LED array in the vu meter-like mouth), two uOLED screens where the robot’s eyes are displayed, and a stereo speaker to generate verbal and non-verbal sounds. Moreover, Mini uses a tablet where videos, images and menus are displayed to ease the interaction with the user.

Next, we detail the configuration of our DMS during this evaluation. All the parameters used in the configuration of the DMS have been decided considering two criteria: first, our previous experience with this type of DMS in robots [36,37,38]; and second, the parameters have been adjusted to obtain a robot that experiences all possibilities for the internal state (i.e., dominant motivation). Before running the evaluation, we empirically fine tuned the DMS until we ended up with a robot where all motivations, sooner or later, became the dominant one.

The configuration of the DMS is a design decision that affects the robot’s behavior. Thus, different values or parameters might end up on a robot exhibiting different behaviors. Studying how the values and the parameters of the DMS affect the robot’s behavior is out of the scope of this work.

### 6.1. State of the Robot

The state of the robot is the combination of its internal state, i.e., the dominant motivation, and its external state, i.e., the state of the robot in relation to the objects/agents that have any influence over the robot, in this case, the user who interacts with it.

#### 6.1.1. Internal State

In this scenario, the robot has been endowed with two motivations, *Social* and *Relax*, which compete between them to become the dominant one. Each motivation is affected by its own drive, defined as *Interaction* and *Rest* respectively (see Table 1). Those drives and motivations have been selected to promote the human-robot interaction and to obtain an animal-like behavior of the robot.

The main objective of the system is to forecast the interaction process with a user, maintaining the wellbeing of the robot as high as possible. Thus, the *Social* motivation has been included to consider the desire of the robot to interact with the user. Its drive, the *Interaction* drive, represents the robot’s need of interaction with people. The *Interaction* drive increases its value in 1 point every 5 s. This drive has a minimum value of 0 points and a saturation value (i.e., its maximum value) of 90 points. The *Interaction* drive is satiated after a direct interaction with the user and, in our scenario, this happens after the robot and the user play a game. The *Social* motivation is affected by an external stimulus: the presence of a user. When the user detection algorithm (see Section 5) perceives the user, this is considered as an incentive and the *Social* motivation rises its value in five points. In other words, the presence of the user around the robot increases its desire of human-robot interaction. Once the *Interaction* drive is satiated, there is a satisfaction time of 60 s before the drive starts, increasing its value again as previously mentioned.

**Figure 5:**
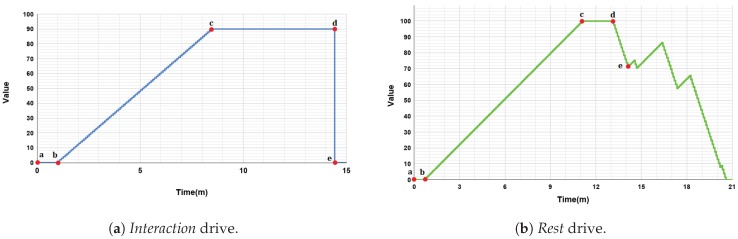
Parametrization and evolution over time of both drives. Interval [a,b) represents the satisfaction time of the drive; [b,c) shows the increase of the drive due to time; [c, d) shows when the drive attains its saturation level; and [d,e) is the satiation of the drive due to the effect of an action (*Play Game* for the drive *Interaction* and *Sleeping* in the case of the drive *Rest*).

In relation to the *Relax* motivation, it has been included to obtain an animal-like behavior of the robot avoiding a hyperactive robot. The *Rest* drive, associated to the *Relax* motivation, increases its value 0.8 points every 5 s excepting when Mini is sleeping, in which case the *Rest* drive decreases 2.4 points every 5 s, three times faster than its increasing speed. Its value ranges from 0 points (minimum value) to 100 points (saturation level). The *Relax* motivation is also affected by external stimuli. In this case, when the robot is alone this motivation rises five points. When the robot detects a user, the incentive disappears. When the *Rest* drive is satiated completely, there is a satisfaction time of 10 s before the drive increases its value again. The evolution of the drives can be observed in Figure 5 and their parameters are summarized in Table 2.

At the beginning of the experiment, both the *Interaction* and *Rest* drives are satiated, that is, their values are 0. When these drives have a value over their own activation level, their motivations can compete to become the dominant motivation. The activation level of the *Interaction* drive has been set to 20 points and the activation level of the *Rest* drive has a value of 10 points. Remember that in case the value of each drive is under its activation level, its corresponding motivation will have a value of 0 points. It is worth noting that, in some cases, it is possible that none of the drives has an intensity over its activation value. In this situation there is non dominant motivation because all Mini’s needs are very low, which can be seen as a very pleasant situation.

#### 6.1.2. External State

The external state of the robot is determined by its relation with external objects or users. In this approach, the external state depends on the influence of the users whose actions have an effect over the robot. Thus, the external state of Mini can be denoted as the state of Mini in relation to the user who interacts with it. In the proposed scenario, the interaction has been limited to a 1-by-1 human-robot interaction. For each user, u_i_, who interacts with the robot, we have three main positions in the environment.

- User is absent: the user is not detected by the robot.
- User is far: the user is detected by the robot, but is not close enough to establish an interaction with it.
- User is near: The user is sit in front of the robot and close enough to interact with it.

Additionally to the positions described above, we have included two transition positions between the *User is far* and *User is near*. Those positions are:

- User is sitting: the user is passing from far to near positions.
- User is standing up: the user is passing from near to far positions.

**Figure 6:**
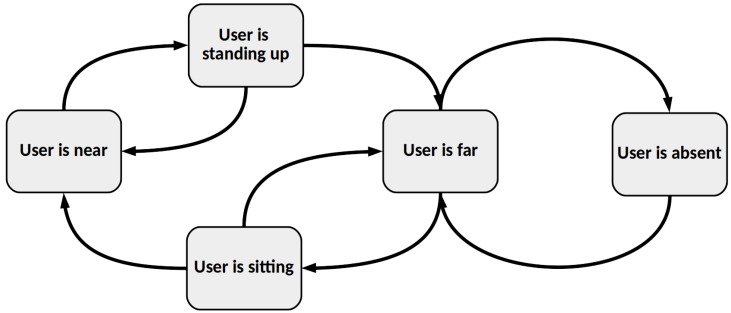
External state of the robot in relation with the position of user in the environment.

The detector explained in Section 5 gives information about if the user is *near*, *far* or *absent*. The positions *User is sitting* and *User is standing up* have been included as a mechanism to let the robot react to two user’s action that affect the robot and the interaction. When the user passes from *User is far* to *User is near* position, Mini updates the position of the user to *User is sitting* and waits 10 s to see if the user really passes to *User is near* or remains in *User is far*. In the same way, when the user passes form *User is near* position to *User is far*, Mini updates the position of the user to *User is standing up* and waits 10 s until the user decides if the user really stands up and passes to *User is far* position or remains in *User is near* position. Hence, Mini can differentiate between five different positions of the user in the environment that are modeled as five different robot’s external states, as shown in Figure 6.

### 6.2. Actions and Effects

The repertoire of actions that Mini can execute in the scenario is oriented to foster HRI and to obtain an animal-like behavior. The actions that Mini is able to perform are:

- Play game: the robot plays an educational game with the user. The game is selected randomly from the repertoire of games.
- Attract attention: Mini uses utterances to convince the user to play with it. For example, Mini says *Someone wants to play with me?*, *There is someone out there?* or *Do not go and play with me*.
- Sleep: Mini rests for a while showing its eyes closed and snoring.

The actions selected by the robot are oriented to satiate the internal needs of the robot, that is, so each action has an effect over the drives. The effect that each action has over the drives are represented in Table 3.

Considering the Sleep action, the value of the *Rest* drive depends on the time the robot has been sleeping. This action has a maximum performing time of 60 s, which will satiate the *Rest* drive reducing it 2.4 points every 5 s, which means that the *Rest* drive is satiated three times faster than it increases. If the robot wakes up before 60 s, the *Rest* drive will diminish its value proportionally to the time the robot has been sleeping. On the other hand, the Attract attention action does not have a direct effect over the drives of the robot, but allows their normal evolution over time. Finally, the Play Game action satiates completely the *Interaction* drive.

It is worth mentioning that an action can be performed just in certain states of the robot because otherwise it does not make sense. For example, in our case, the action Play Game can only be performed if the *User is near* position.

### 6.3. Method

**Figure 7:**
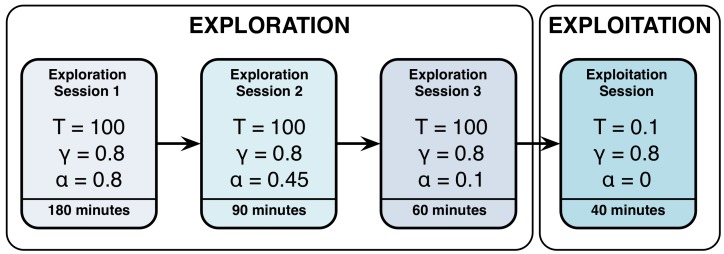
Phases of the experiment where the DMS has been tested.

The evaluation is composed of two phases: (i) exploration and (ii) exploitation (see Figure 7). During the exploration, the robot goes through all possible states and tries all possible actions. Thanks to the Q-learning algorithm [32], Mini learns how good it is to perform each action in each state. Q-learning algorithm assigns a Q-value to every <state, action> pair. At the beginning, each Q-value is set to the same value, considering that the robot learns from scratch.

In this experiment, the exploration phase has been divided into three sessions of 180, 90, and 60 min respectively. The duration of each stage of the exploration phase has been set empirically based on previous works [36,37,38] where we observed the required time to explore all <state, action> pairs a sufficient number of times.

During these sessions the actions were randomly selected using the Boltzmann equation [39] with a temperature T=100. Randomness in the action selection allows to explore all actions in all states updating its corresponding Q-values. The discount factor γ was set to 0.8 points, giving a great significance to the future rewards during the exploration phase. The learning rate α started at a high value of 0.8 during the first exploration session and then decreased to 0.45 and 0.1 in the two subsequent sessions; this meant that as long as we progressed in the learning phase, we increased the importance of previously learned information.

When the learning phase was completed, we evaluated the learned policy of actions in the exploitation phase. In this phase, learning is blocked by setting the learning rate α to 0 and the actions are selected based on their Q-values: the higher the value, the more chances to be selected. This was achieved by reducing the temperature parameter in Boltzmann’s equation to 0.1. During the 40-min exploitation phase, Mini visited all states and selected the actions with the highest Q-values taking into account the state of the robot in relation with the user. The results are described in Section 7.

In this experiment, a voluntary student interacted with Mini during both the exploration and exploitation phases.

The robot was placed on a table in the laboratory. An experimenter introduced the robot and asked the user to interact on his own will during the sessions. The experimenter informed that he could move freely in the room and, when he wanted to interact with Mini, he had to sit down in front of the robot. The student was not informed about the experiment or its goals.

The user reacted to the robot’s actions as follows: when the robot was sleeping, the user performed random actions, e.g., approaching or moving away from the robot; when the robot called the attention of the user, he approximated it; and if the robot decided to play a game, the user normally played with the robot. It is worth noting that this behavior was the one exhibited by the user most of the time but he behaved at his convenience.

## 7. Results

In this section we present the results obtained during the exploitation phase. We have considered two types of results: the learned behaviors and the robot’s wellbeing.

### 7.1. Learned Behaviors

**Figure 8:**
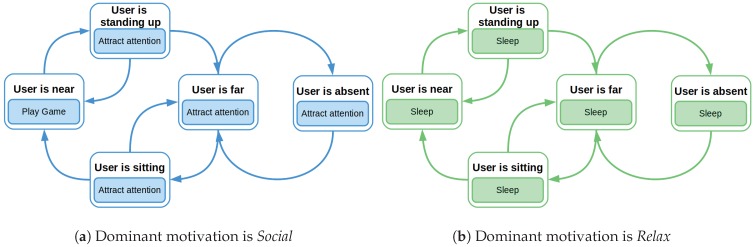
Policies learned by the robot with the different dominant motivations.

After the learning process, the robot has to exploit the acquired knowledge. Figure 8 presents the policy of actions that Mini has learned during the exploration phase. It contains the best actions, i.e., the actions with the highest Q-values, in every state. If the dominant motivation is *Social* (Figure 8a), Mini has learned that when the *User is near*, it has to execute the *Play Game* action. This is the best one because it satiates the need of interaction. In other states, *Attract attention* is the best action because it causes the user to approach Mini and eventually they play together. Once the robot is in the *User is near* external state, the robot decides to play the game with the user. Focusing on the *Relax*, when this is the dominant motivation, Mini has learned that it has to sleep no matter where the user is (Figure 8b). This action is the only one that reduces the need for rest, which is the drive related to the *Relax* motivation.

It is important to mention that these behaviors have been learned through the interaction with one user considering his reactions to the robot actions. Some of these actions depend on the user. This is the case of the action “play game” that requires a user close to the robot and this depends on his/her behavior. When this action needs to be executed (in our case when the dominant motivation is Social), the robot learns the sequence of actions to get the user closer before they play together; in this case by executing the action “attract attention”. If the behavior of the user would have deviated significantly from the one detailed in Section 6.3, the policy learned by the robot might have been different, leading to different robot behaviors in the exploitation phase. For example, if the user had approached the robot just when it was sleeping (maybe because he/she did not like the “attract attention” action), the robot might have learned to sleep until the user sits in front of the robot and then they play a game. This could happen also if we consider multiple users who behave disparately. For instance, if a user does not want to interact with the robot and never plays with it, in this case, the robot might learn that the best action would be to sleep most of the time. The evaluation of multiple policies according to different users is out of the scope of this paper.

### 7.2. Mini’s Wellbeing

**Figure 9:**
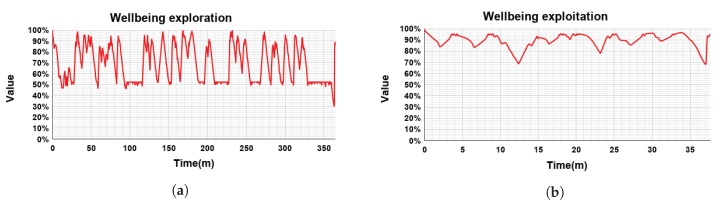
Evolution of Mini’s wellbeing during the evaluation.

Considering that the robot’s wellbeing has been employed in the reward signal, when exploiting the learned policy we are maximizing the reward, and consequently the wellbeing. This can be observed when comparing the wellbeing during the exploitation and the exploration (Figure 9). As it was explained in previous sections, the wellbeing is represented as a percentage where 100% means the robot has all its drives satiated ($Wb_{ideal}$) and 0% represents the situation when all its drives have reached its maximum value.

During the exploration phase, actions were selected randomly and therefore the wellbeing fluctuated greatly (Figure 9a). Looking at Figure 9a, the robot’s wellbeing stays over 50% most of the time, although sometimes its value drops to 30%. In this phase, i.e., during learning, the wellbeing has a mean value of 67.65% and a standard deviation of 17.35%.

In the exploitation phase, the robot selected the best action in order to reduce its drives as much as possible, that is, to maximize its wellbeing. Thus, the wellbeing of the robot should remain in an optimal value almost always. Figure 9b shows the wellbeing of the robot during the exploitation phase. The wellbeing is almost always above the 80%, having a mean value of 90.35% and a standard deviation of 5.78%. This stable high level of wellbeing represents that the learned policy was appropriate and the needs of the robot stayed very low.

Comparing both the exploration and exploitation phases, we can say that the average robot’s wellbeing in the exploitation phase is more than 20 points over the average wellbeing in the exploration phase. In addition, the standard deviation in the exploration phase is three times higher than its corresponding value in the exploitation phase (17.35% vs. 5.78% respectively), which means that the wellbeing during the exploration phase has a much bigger variation than during the exploitation phase. In other words, when exploiting the learned policy of action, Mini’s wellbeing is more stable than when learning.

## 8. Conclusions

In this paper, we have presented the biological concepts that have inspired our decision-making system where drives and motivations govern the robot’s behavior. We have shown how those concepts can be applied to a DMS integrated in a social robot. The proposal has been evaluated in a realistic HRI scenario. In this scenario the robot has learned how to keep its wellbeing as high as possible by following the policy of actions that depends on the dominant motivation (internal state) and on the position of the user in relation with the robot (external state).

We have presented in this manuscript a simple robust user detector using the onboard RGB-D camera. The algorithm is able to establish when a user is ready for interaction, through a body detector based on depth information, or far from the robot, using the colour images to detect users’ faces. The detector takes into account the body occlusions that occur when the user is near to the camera.

The evaluation of our DMS was made in two phases. During the exploration phase the robot learned which action was the best option for every state using the Q-learning algorithm. The duration of the exploration phase was set to 330 min approximately to let the robot enough time to learn the correct behavior in each situation. Afterwards, the performance of the learned behaviors was tested in the 40-min exploration phase.

Our evaluation showed that including the motivations *Social* and *Relax* we obtained a proactive robot exhibiting an animal-like behavior: when it is motivated to socialize (dominant motivation is *Social*), first, it calls the attention of the user, if (s)he is not close enough, and then they play games together; in case of the robot’s dominant motivation is *Relax*, it slept. Comparing the robot’s wellbeing in both phases, as expected, during the exploitation phase the wellbeing was significantly higher than during the exploration phase. In the exploration phase, the wellbeing had a mean value of 67.65% (σ=17.35%). When the robot exploited the learned policy, the wellbeing presented a mean value of 90.35% (σ=5.78%).

It is important to mention that the robot learns its behavior as a response to a user acting on its own will during the exploration phase. Therefore, for users behaving in different manners during the learning phase, the resulting policies would adapt accordingly. For this reason, if a robot implementing our DMS had to interact with multiple users, the decision-making process could consider a different policy per user. The evaluation of different robots’ behaviors depending on several users will be part of future works.

## Figures and Tables

**Table 1:** Relationship between each drive and its motivation.

Drive	Motivation
Interaction	Social
Rest	Relax

**Table 2:** Drives parameters configurations.

Drive	Value Range	Satisfaction Time (s)	Increased by	Decreased by
Interaction	[0–90]	60	1 points/5 s	Play game
Rest	[0–100]	10	0.8 points/5 s	Sleep

**Table 3:** Effect of each action over the drives of the robot.

Action	Drive Affected	Value
Sleep	Rest	−2.4 points/5 s
Attract attention	None	None
Play Game	Interaction	set to 0 points

## References

[1] Magrini E., De Luca A. Human-robot coexistence and contact handling with redundant robots. Proceedings of the 2017 IEEE/RSJ International Conference on Intelligent Robots and Systems (IROS).

[2] Sciutti A., Mara M., Tagliasco V., Sandini G. (2018). Humanizing Human-Robot Interaction: On the Importance of Mutual Understanding. IEEE Technol. Soc. Mag..

[3] Pfeifer R., Lungarella M., Iida F. (2012). The Challenges Ahead for Bio-inspired ‘Soft’ Robotics. Commun. ACM.

[4] Pfeifer R., Lungarella M., Iida F. (2007). Self-Organization, Embodiment, and Biologically Inspired Robotics. Science.

[5] Kandel E., Schwartz J., Jessell T. (1991). Principles of Neural Science.

[6] Veldhuis A. (2011). Reviewing Decision Making: From Awareness to Social Decision Making. Master’s Thesis.

[7] Bear M., Connors B., Paradiso M. (2001). Neuroscience: Exploring the Brain.

[8] Berridge K.C. (2004). Motivation concepts in behavioral neuroscience. Physiol. Behav..

[9] Hull C.L. (1943). Principles of Behavior: An Introduction to Behavior Theory.

[10] Cherry K. Drive-Reduction Theory and Human Behavior Biological Need Motivates Behavior. https://www.verywellmind.com/drive-reduction-theory-2795381.

[11] Hull C. (1935). The conflicting psychologies of learning—A way out. Psychol. Rev..

[12] Schultz D.P., Schultz S.E. (2005). A History of Modern Psychology.

[13] Santa-Cruz J., Tobal J.M., Vindel A.C., Fernndez E.G. (1989). Introduccin a La Psicologa.

[14] Olds J., Milner P. (1954). Positive reinforcement produced by electrical stimulation of septal area and other regions of rat brain. J. Comp. Physiol. Psychol..

[15] Deutsch J., Howarth C. (1963). Some tests of a theory of intracranial self-stimulation. Psychol. Rev..

[16] Velásquez J.D. Modeling Emotions and Other Motivations in Synthetic Agents. Proceedings of the Fourteenth National Conference on Artificial Intelligence.

[17] Arkin R.C., Ali K., Weitzenfeld A., Cervantes-Perez F. (2000). Behavioral models of the praying mantis as a basis for robotic behavior. Robot. Auton. Syst..

[18] Arkin R.C., Fujita M., Tagaki T., Hasegawa R. An Ethological and Emotional Basis for Human- Robot Interaction. Proceedings of the IEEE/RSJ International Conference on Intelligent Robots and Systems (IROS 2002).

[19] Stoytchev A., Arkin R.C. (2004). Incorporating Motivation in a Hybrid Robot Architecture. J. Adv. Comput. Intell. Intell. Inform..

[20] Cañamero D. (2003). Designing Emotions for Activity Selection. Emotions in Humans and Artifacts.

[21] Canamero D. (1997). A hormonal model of emotions for behavior control. VUB AI-Lab Memo.

[22] Breazeal C.L. (2004). Designing Sociable Robots.

[23] Parisi D. (2004). Internal robotics. Connect. Sci..

[24] Vouloutsi V., Lallée S., Verschure P.F.M.J. (2013). Modulating behaviors using allostatic control. Lect. Notes Comput. Sci..

[25] Cao H.L., Gómez Esteban P., Albert D.B., Simut R., Van de Perre G., Lefeber D., Vanderborght B. (2017). A Collaborative Homeostatic-Based Behavior Controller for Social Robots in Human—Robot Interaction Experiments. Int. J. Soc. Robot..

[26] Hieida C., Horii T., Nagai T. (2018). Decision-Making in Emotion Model. Proceedings of the Companion of the 2018 ACM/IEEE International Conference on Human-Robot Interaction.

[27] Balkenius C. (1995). Natural Intelligence in Artificial Creatures. Ph.D. Thesis.

[28] Avila-Garcia O., Cañamero L. Using Hormonal Feedback to Modulate Action Selection in a Competitive Scenario. Proceedings of the 8th International Conference on Simulation of Adaptive Behavior (SAB’04).

[29] Lorenz K., Leyhausen P. (1973). Motivation of Human and Animal Behaviour; An Ethological View.

[30] Blumberg B.M., Todd P.M., Maes P. (1996). No Bad Dogs: Ethological Lessons for Learning in Hamsterdam.

[31] Cañamero L. (1997). Modeling Motivations and Emotions as a Basis for Intelligent Behavior. Proceedings of the First International Symposium on Autonomous Agents (Agents’97).

[32] Sutton R.S., Barto A.G., Bach F. (1998). Reinforcement Learning: An Introduction.

[33] NASA Man-Systems Integration Standards. https://msis.jsc.nasa.gov/sections/Section03.htm.

[34] Viola P., Jones M.J. (2004). Robust real-time face detection. Int. J. Comput. Vis..

[35] Alonso-Martin F., Castro-Gonzalez A., Gorostiza J.F., Salichs M.A., Herrmann G., Pearson M.J., Lenz A., Bremner P., Spiers A., Leonards U. (2013). Multidomain Voice Activity Detection during Human-Robot Interaction. Social Robotics. ICSR 2013. Lecture Notes in Computer Science.

[36] Castro-Gonzalez A., Malfaz M., Salichs M.A. (2011). Learning the Selection of Actions for an Autonomous Social Robot by Reinforcement Learning Based on Motivations. Int. J. Soc. Robot..

[37] Castro-Gonzalez A., Malfaz M., Salichs M.A. (2013). An Autonomous Social Robot in Fear. IEEE Trans. Auton. Mental Dev..

[38] Castro-Gonzalez A., Malfaz M., Gorostiza J.F., Salichs M.A. (2014). Learning Behaviors by an Autonomous Social Robot with Motivations. Cybern. Syst..

[39] Cercignani C. (1988). The boltzmann equation. The Boltzmann Equation and Its Applications.

